# Identification, Characterization, and In Vitro Fungicide Sensitivity of *Paraphoma radicina*, the First Reported Cause of Root Rot in *Polygonatum sibiricum*

**DOI:** 10.3390/jof11110770

**Published:** 2025-10-26

**Authors:** Wenchao Li, Qingyi Zhao, Jin Zhang, Shuyuan Mu, Yangyang Sun, Shuangjiao Ma, Chengming Yu, Yehan Tian, Huixiang Liu

**Affiliations:** 1 College of Plant Protection, Shandong Agricultural University, Tai’an 271018, China; 2 Taishan Academy of Forestry Sciences, Tai’an 271000, China; 3 College of Forestry, Shandong Agricultural University, Tai’an 271018, China

**Keywords:** *Polygonatum sibiricum*, *Paraphoma radicina*, root rot, fungicide sensitivity

## Abstract

*Polygonatum sibiricum*, a perennial medicinal and edible herb, has recently suffered severe root rot outbreaks in China, threatening its yield and quality. From 2023 to 2024, plants showing rhizome darkening and necrosis were observed in Tai’an, Shandong Province. A fungal isolate (HJ2B3) was consistently recovered from symptomatic tissues and confirmed as pathogenic through in vitro detached rhizome and potted plant inoculations. The isolate produced globose to subglobose, setose pycnidia and hyaline, aseptate conidia. Morphological characteristics, combined with *ITS*, *EF1-α*, and *β-tubulin* sequence analyses and phylogenetic evidence, identified the pathogen as *Paraphoma radicina*. In vitro fungicide assays using the mycelial growth rate method showed that difenoconazole, prochloraz, and pyraclostrobin were the most effective, with EC_50_ values of 0.06, 0.32, and 0.36 mg/L, respectively, whereas tetramycin and azoxystrobin exhibited lower inhibitory activity. This study reports *P. radicina* as the causal agent of *P. sibiricum* root rot for the first time, expands the known host range of this pathogen, and provides the first baseline data on its in vitro sensitivity to five fungicides. These results offer practical guidance for chemical control and further evaluation under greenhouse or field conditions in *P. sibiricum* cultivation.

## 1. Introduction

*Polygonatum sibiricum*, a traditional medicinal and edible plant in the family Asparagaceae, possesses rhizomes rich in polysaccharides, saponins, flavonoids, and alkaloids, which exhibit antioxidant, immunomodulatory, anti-osteoporotic, and hypoglycemic activities [1,2,3]. Due to its diverse pharmacological properties, *P. sibiricum* has been widely utilized in traditional Chinese medicine, functional foods, and nutraceuticals [4]. With increasing health awareness, its cultivation area in China has expanded rapidly; however, this growth has been accompanied by rising disease incidence, particularly root rot, which now poses a major constraint to sustainable production.

Root rot of *P. sibiricum* is a destructive soil-borne disease prevalent across major cultivation regions in China [5]. The disease is caused by a complex of pathogens that vary geographically, with fungi, bacteria, and nematodes all involved; among these, fungi are the predominant causal agents. Previous studies have identified several fungal genera, including *Fusarium*, *Rhizoctonia*, and *Pythium*, with *Fusarium* species dominating more than 70% of the isolates [6]. Species such as *F. oxysporum*, *F. solani*, *F. foetens*, *F. hostae*, and Colletotrichum spaethianum have been confirmed to induce typical root rot symptoms in *Polygonatum* spp. [7,8,9]. Increasing evidence suggests that root rot of *Polygonatum* plants often results from mixed infections involving multiple pathogens, greatly complicating disease management [10].

Although root rot is commonly associated with *Fusarium* and other well-known fungi, additional pathogenic fungi may also contribute to disease incidence. Recent studies suggest that some previously overlooked genera could play a role in disease development, highlighting the need for a more comprehensive investigation of the pathogens affecting *P. sibiricum*. Among these, the genus *Paraphoma*—a soil-borne fungus capable of infecting multiple hosts—warrants further study for its potential involvement in root rot of *P. sibiricum.*

The genus *Paraphoma* has undergone several taxonomic revisions. It was established by Morgan-Jones and White in 1983 based on setose conidiomata in the type species *P. radicina*, distinguishing it from *Phoma*. Later, it was treated as a section within *Phoma*, but multi-gene phylogenetic analyses led to its elevation to genus level within Didymellaceae [11]. The genus is species-rich, though the sexual states of most species remain unclear, making it important in fungal taxonomy and plant pathology. Molecular markers such as *ITS*, *LSU*, *SSU*, *EF1-α*, *RPB2*, and *β-tubulin* (*TUB*) are widely used for identification. Using these markers and morphology, [12] identified *P. dioscoreae* on *Dioscorea tokoro*; [13] reported *P. vinacea* causing crown rot on *Tanacetum cinerariifolium*; and [14] described *P. chlamydocopiosa* and *P. pye* infecting leaves and crowns of the same host. Currently, seven species are recognized: *P. radicina*, *P. chrysanthemicola*, *P. fiemti*, *P. dioscoreae*, *P. vinacea*, *P. chlamydocopiosa*, and *P. pye*.

*Paraphoma* species are mainly soil-borne pathogens causing root and crown rot on diverse hosts across Australia, the Americas, and Eurasia [14]. *P. radicina* was first isolated from European sour cherry in Australia in 1901, later reported on European wild apple and tomato [11]. In China, it was first recorded from diseased cysts of soybean cyst nematode [15] and can infect alfalfa roots, causing root rot [16]. Other species, such as *P. chrysanthemicola*, infect chrysanthemum and alfalfa roots and cause leaf spot on Atractylodes [17,18,19], while *P. vinacea*, *P. pye*, and *P. chlamydocopiosa* are pathogens of Australian Tanacetum species [13,14].

Chemical control remains the main strategy for managing root rot; however, strict pesticide residue limits in medicinal plants require fungicides that are highly effective, low in toxicity, and leave minimal residues. Studies on medicinal herbs have identified several fungicides with strong activity against root rot pathogens. Tetramycin inhibits mycelial growth and spore germination of *Fusarium oxysporum*, *F. solani*, and *Cylindrocarpon destructans* in a dose-dependent manner [20,21]. Difenoconazole shows high antifungal activity against *Fusarium* isolates from Scutellaria baicalensis and Bletilla striata, outperforming carbendazim, azoxystrobin, and other fungicides [22,23]. Pyraclostrobin and prochloraz also demonstrate substantial inhibition of key root rot pathogens, including seed-associated fungi, whereas azoxystrobin shows moderate efficacy depending on pathogen and concentration [24,25]. In *Polygonatum* species, tetramycin, difenoconazole, and prochloraz effectively suppress dominant pathogens such as *F. solani* and *F. oxysporum*, and combined applications can further enhance control efficiency [26,27]. These studies provide a basis for developing targeted chemical control strategies for root rot in *P. sibiricum.*

Despite these advances, information on other potential pathogenic fungi associated with *P. sibiricum* root rot remains limited. Moreover, chemical control options targeting newly emerging pathogens have not been systematically evaluated. Therefore, the objectives of this study were to (1) identify and characterize the primary pathogenic fungus causing root rot of *P. sibiricum* using three molecular markers (*ITS*, *EF1-α*, and *TUB*) combined with morphological analysis and in vitro inoculation assays to confirm pathogenicity, and (2) evaluate the in vitro efficacy of five representative fungicides: tetramycin, difenoconazole, prochloraz, pyraclostrobin, and azoxystrobin. This study expands current knowledge of the pathogenic fungi diversity associated with *P. sibiricum* root rot and provides a foundation for improved diagnosis and development of targeted management strategies against this emerging disease.

## 2. Materials and Methods

### 2.1. Sampling

From May to July 2024, a total of 12 *P. sibiricum* plants exhibiting typical root rot symptoms were collected from Mount Tai, Shandong Province, China. The major disease symptoms included chlorosis and wilting of stems and leaves, as well as browning to black necrosis of root tissues.

### 2.2. Chemicals

The following fungicides were used in this study: 0.3% tetramycin (AS; Wkioc Bioengineering Co., Ltd., Liaoning, China), 40% difenoconazole (SC; Dongtai Agrochemical Co., Ltd., Liaocheng, Shandong, China), 30% pyraclostrobin (SC; Haina Biological Technology Co., Ltd., Qingdao, China), 450 g/L prochloraz (EW; Haina Biological Technology Co., Ltd., Qingdao, China), and 250 g/L azoxystrobin (SC; Obes Biochemical Technology Co., Ltd., Yantai, China). Genomic DNA was extracted using a Fungi Genomic DNA Extraction Kit (Solarbio science & technology Co., Ltd., Beijing, China).

### 2.3. Pathogen Isolation

Pathogen isolation was conducted following the method described by Zhou et al. [28]. Diseased rhizomes were first rinsed with sterile water to remove surface debris. Tissue blocks (5 mm × 5 mm) were excised from the junction between healthy and diseased tissue, surface-sterilized in 75% ethanol for 30 s, treated with 1% sodium hypochlorite for 1 min, and rinsed three times with sterile distilled water. Sterilized tissue blocks were blotted dry and placed on potato dextrose agar (PDA) plates, which were incubated in the dark at 25 °C for 5 days. Hyphal tips were transferred to fresh PDA to obtain pure cultures. After 21 days, the isolate HJ2B3 was selected, and temporary slides were prepared to observe colony morphology and spore characteristics under a microscope. The isolated strain was preserved as a mycelial plug in 20% glycerol at −80 °C and cultured on different media (Supplementary Table S1) to observe colony morphology and growth characteristics.

### 2.4. Molecular Identification

Genomic DNA was extracted using the Fungi Genomic DNA Extraction Kit (Solarbio Life Sciences, Beijing, China). Three loci were amplified: the rDNA internal transcribed spacer (*ITS*), translation elongation factor 1-α (*EF1-α*) and *β-tubulin* (*TUB*), using primer pairs ITS1/ITS4, TuB2Fd/TuB4Rd, and EF1-728F/EF2, respectively (Table 1). PCR was performed in a 25 μL reaction volume containing 1 μL of each primer, 2 μL of DNA template, 12.5 μL of 2× Taq PCR Master Mix, and 8.5 μL of sterile distilled water. The cycling program consisted of an initial denaturation at 95 °C for 5 min, followed by 40 cycles of 95 °C for 30 s, annealing at 48 °C (ITS1/ITS4) or 52 °C (TuB2Fd/TuB4Rd, EF1-728F/EF2) for 30 s, and extension at 72 °C for 80 s, with a final extension at 72 °C for 7 min [12,29].

PCR products were stored at 4 °C and sequenced bidirectionally at Sangon Biotech Co., Ltd. (Shanghai, China). Consensus sequences were assembled using DNAman version 5.2.2 (LynnonBiosoft) and deposited in GenBank. For phylogenetic analysis, *ITS*, *EF1-α* and *TUB* sequences from this study and reference sequences retrieved from GenBank (Table 2) were aligned [13,14], with *Neosetophoma samarorum* designated as the outgroup. Neighbor-joining (N-J) phylogenetic trees were constructed in MEGA11.0 (Molecular Evolutionary Genetics Analysis; Sudhir Kumar, Temple University, Philadelphia, PA, USA) [34].

### 2.5. Pathogenicity Assays

Pathogenicity assays were conducted on both excised rhizomes and potted *P. sibiricum* seedlings. Healthy rhizomes were surface-sterilized with 75% ethanol and wounded with a sterile needle. Mycelial plugs (5 mm in diameter) from PDA cultures of ten isolates were placed on wound sites, while sterile PDA plugs served as controls. Inoculated rhizomes were incubated at 25 °C and 90% relative humidity in the dark for 10 days, with symptom development monitored daily. To further confirm pathogenicity, the most virulent isolate (HJ2B3) was inoculated onto healthy seedlings under greenhouse conditions. Conidial suspensions were prepared by flooding sporulating PDA cultures with 10 mL sterile water, scraping the colony surface, filtering through sterile gauze, and adjusting the concentration to 1 × 10^6^ conidia/mL using a hemocytometer. Uniform seedlings were selected, and 15 mL of suspension was applied to the rhizome region of each plant; sterile water served as the control. Each treatment was repeated three times. Seedlings were transplanted into sterilized soil and maintained in a greenhouse for two months. Fungi were re-isolated from symptomatic tissues and identified morphologically and molecularly, fulfilling Koch’s postulates.

### 2.6. In Vitro Toxicity Bioassay of Chemical Agents

A 7 mm mycelial plug of the pathogenic isolate was inoculated onto PDA plates amended with different concentrations of 5 chemical agents (Table 3). The plates were incubated at 28 °C in the dark, with three replicates per treatment. After 5 days, colony diameters were measured using the cross-measurement method. The inhibitory effect of each agent on mycelial growth was determined using the mycelial growth rate method, according to the following Formula (1).

(1) $$\mathrm{Mycelial}\;\mathrm{growth}\;\mathrm{inhibition}\;\mathrm{rate}\;(\%)=\;\frac{\mathrm{Colony}\;\mathrm{diameter}\;\mathrm{of}\;\mathrm{control}-\mathrm{Colony}\;\mathrm{diameter}\;\mathrm{of}\;\mathrm{treatment}}{\mathrm{Colony}\;\mathrm{diameter}\;\mathrm{of}\;\mathrm{control}-\mathrm{Diameter}\;\mathrm{of}\;\mathrm{initial}\;\mathrm{plug}}\;\times \;100$$

The fungicidal activities of five selected fungicides against *P radicina* strain HJ2B3 were assessed using the mycelial growth rate method. The inhibition rates at varying fungicide concentrations were transformed into probit values (y), and the log-transformed fungicide concentrations (x) were used as the independent variable. A linear regression equation of the form y = a + bx was established using the least squares method. The EC_50_ (mg·L^−1^) was derived from the regression equation as the concentration corresponding to y = 5 [35]. The coefficient of determination (R^2^) was used to evaluate the goodness of fit of the regression model. Based on the EC_50_ values, the inhibitory efficacy of different fungicides against *P. radicina* was compared. All statistical analyses and regression modeling were performed using IBM SPSS Statistics version 26.0 (IBM Corp., Armonk, NY, USA).

## 3. Results

### 3.1. Pathogen Isolation and Morphological Characteristics

**Figure 1:**
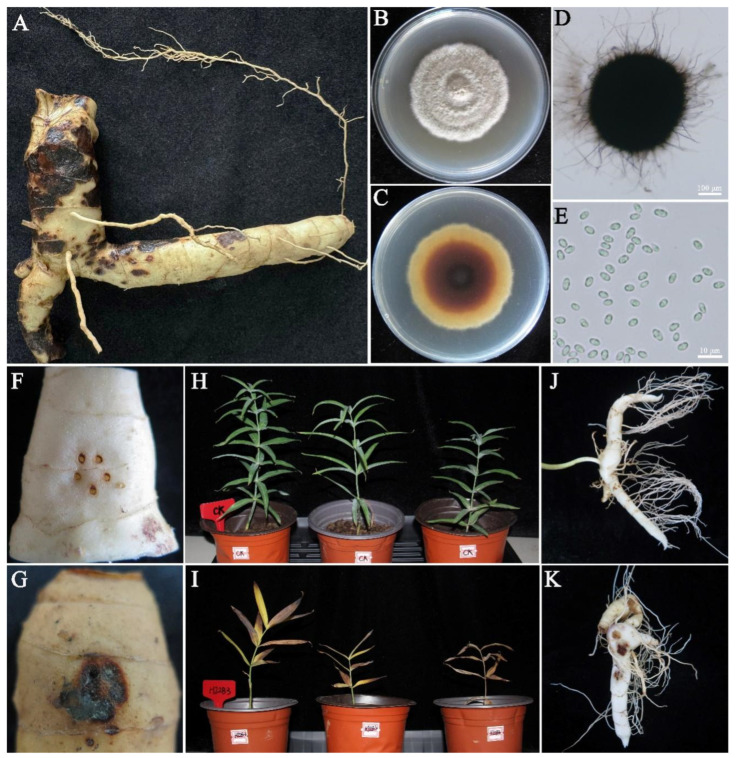
Morphological characteristics of *Paraphoma radicina* HJ2B3: (**A**). Isolation from diseased rhizome; colony morphology on PDA after 21 days in front (**B**) and back (**C**); pycnidium (**D**); conidia (**E**). Symptoms on detached tuberous rhizomes (**F**,**G**) and potted plants (**H**–**K**) ten days post-inoculation.

During the 2023–2024 growing seasons, recurrent outbreaks of root rot were observed on *P. sibiricum* at the Taishan *P. sibiricum* Research Station (Tai’an, Shandong Province, China). Field investigations revealed that the disease primarily affected the roots and stem bases, typically initiating from the rhizomes. As subterranean rhizome lesions expanded, the root system exhibited characteristic water-soaked, rotting symptoms, which progressed to complete decay in severe cases. Notably, a distinctive symptom pattern was observed: initial symptoms manifested as dark brown lesions on the rhizome epidermis, which gradually enlarged and resulted in black necrotic roots. Through tissue isolation, a predominant fungal strain, designated HJ2B3, was consistently recovered from infected rhizome tissues (Figure 1A). On PDA, HJ2B3 developed dense aerial mycelia, with colonies exhibiting color variations from gray to off-white, typically bordered by distinct rings of white hyphae (Figure 1B,C). The colony reverse showed a pale-yellow periphery, with pigmentation intensifying toward the center. After 4 weeks of incubation at 25 °C in the dark, mature colonies turned gray-green and produced numerous black granular pycnidia on the agar surface. Microscopic examination revealed that pycnidia were globose to subglobose, 341–458 μm in diameter (mean = 396 μm), with variable ostiolar papillae, and covered with dense setae averaging 246.3 μm in length (Figure 1D). Abundant conidia released by gentle crushing in water mounts were hyaline, aseptate, and ellipsoidal to subglobose, with dimensions of 3.4–5.4 × 1.8–3.3 μm (Figure 1E). Based on preliminary morphological characteristics, the isolate was initially classified as a member of the genus *Paraphoma* (*Paraphoma* sp.), consistent with previous reports on other plant pathogenic species in this genus [16]. The colony morphology of HJ2B3 on different media is shown in Supplementary Figure S1.

Pathogenicity assays showed that HJ2B3 induced typical root rot symptoms on detached *P. sibiricum* rhizomes, including tissue discoloration and decay, whereas no symptoms developed in sterile water controls (Figure 1F,G). Seven days after inoculation, all potted plants developed root rot symptoms, characterized by rhizome blackening and decay, consistent with field observations (Figure 1I,K). No symptoms were observed in control plants (Figure 1H,J). The fungus re-isolated from symptomatic rhizomes of inoculated plants was morphologically identical to HJ2B3. Subsequent molecular and morphological analyses confirmed its identity, thereby fulfilling Koch’s postulates and verifying HJ2B3 as the causal agent of *P. sibiricum* root rot.

### 3.2. Molecular Identification and Phylogenetic Analysis

**Figure 2:**
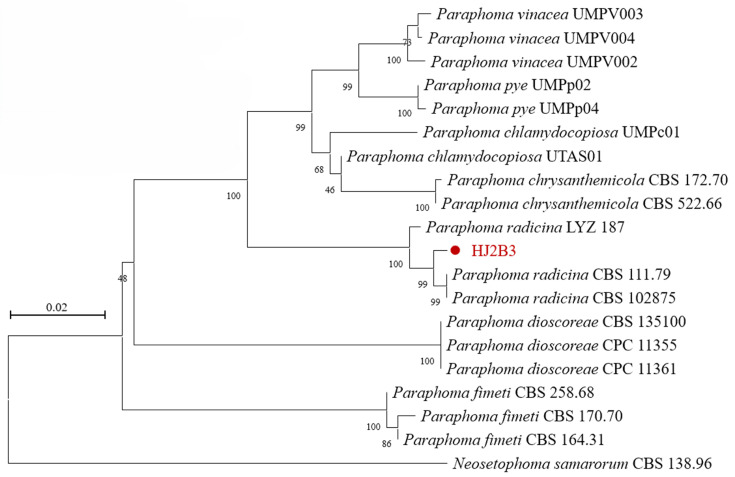
Neighbor-joining phylogenetic tree based on concatenated *ITS*, *EF1-α*, and *TUB* sequences, showing the relationship of HJ2B3 (highlighted in red font and marked with red dots) with reference *Paraphoma* species.

The sequencing data were assembled and edited using DNAman v5.2.2. Three primer pairs successfully amplified clear fragments corresponding to the *ITS*, *EF1-α*, and *TUB* genes. The obtained sequences have been deposited in the NCBI GenBank database under accession numbers PX123865 (*ITS*, 539 bp), PX128137 (*EF1-α*, 593 bp), and PX128136 (*TUB*, 347 bp). Based on sequence similarity and relevant taxonomic literature, 18 reference strains of *Paraphoma* with high sequence homology were selected from GenBank. These sequences, along with HJ2B3, were concatenated in the order *ITS*–*EF1-α*–*TUB* to construct a multi-locus phylogenetic tree. Phylogenetic analysis using the neighbor-joining (NJ) method on the concatenated sequences showed that strain HJ2B3 clustered with two ex-type strains of *P. radicina* (CBS 102875 and CBS 111.79), forming a monophyletic group with 99% bootstrap support (Figure 2). By integrating phenotypic (cultural and morphological) and molecular evidence, strain HJ2B3 was identified as *P. radicina*.

### 3.3. Inhibitory Efficacy of Fungicides Against P. radicina

**Figure 3:**
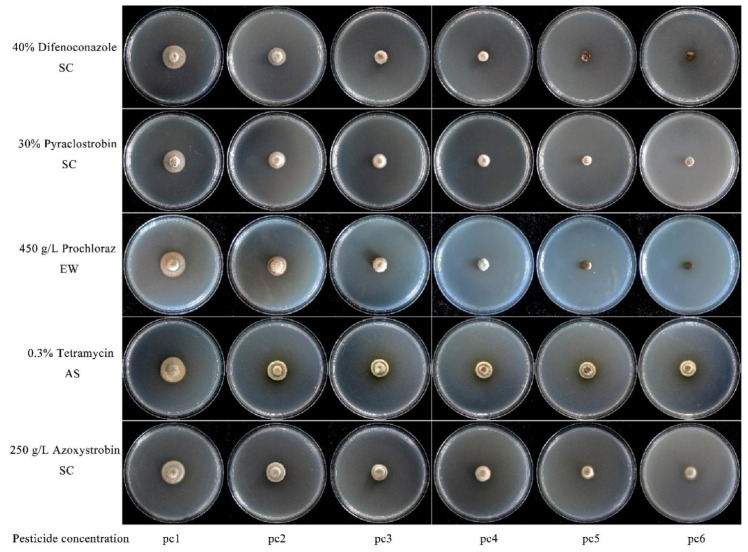
Fungicidal activity of the five tested compounds against HJ2B3.

The five fungicides tested exhibited significant inhibitory activity against HJ2B3 (Table 4, Figure 3). Difenoconazole, prochloraz, and pyraclostrobin were the most effective, with half-maximal effective concentrations (EC_50_) of 0.0640, 0.3253, and 0.3602 mg/L, respectively. Tetramycin displayed moderate activity (EC_50_ = 2.1020 mg/L), whereas azoxystrobin was the least effective (EC_50_ = 5.4280 mg/L). High R^2^ values (0.9418–0.9788) for the regression equations confirmed the reliability of the dose–response relationships. These results revealed marked variations in fungicidal activity among the five fungicides, with difenoconazole demonstrating the strongest inhibition against HJ2B3. The findings provide a scientific basis for selecting effective fungicides for controlling root rot of *P. sibiricum* root rot caused by *P. radicina*.

## 4. Discussion

This study establishes *P*. *radicina* as a novel causal agent of root rot in *P*. *sibiricum*, marking a significant advancement in understanding disease threats to this valuable medicinal crop. The identification, confirmed through morphological, molecular (*ITS*, *EF1-α*, and *TUB*), and pathogenicity analyses, expands the known host range of *P. radicina* and highlights its ability to infect multiple hosts.

Recent studies on *Paraphoma* species have revealed their ecological versatility: they function not only as saprophytes but also as significant pathogens, with certain species capable of existing as endophytes in symbiotic association with their host plants. Fungi of this genus were initially identified in Australia, the United States, and Europe as soil-borne pathogens responsible for root and collar rot in various hosts [13]. Further investigations have demonstrated that *Paraphoma* species can infect the leaves of Atractylodes japonica, causing leaf spot [19], as well as the root collar of Tanacetum cinerariifolium, leading to collar rot [13,14]. *P*. *radicina*, the type species of the genus, was first reported on sour cherry (*Prunus cerasus*) in Australia, and has subsequently been isolated from wild apple (*Malus sylvestris*) in the Netherlands and tomato (*Solanum lycopersicum*) in Germany [11], reflecting its wide host range and potential as a globally distributed plant pathogen.

The identification of plant pathogenic fungi mainly relies on two complementary approaches: traditional morphological observation and molecular (phylogenetic) analysis. Morphological identification can be affected by many environmental factors, which may lead to inconsistent or even misleading results. Therefore, combining morphological features with molecular evidence usually provides the most reliable basis for fungal identification [36]. Before 2004, the taxonomy of the genus *Phoma* was largely based on morphology. [37] proposed nine groups within the genus, but the boundaries among groups and between individual species were often unclear. Moreover, this classification did not accurately reflect the evolutionary relationships within the group [29]. With the progress of molecular biology, researchers have gradually adopted DNA sequence data to clarify fungal systematics. In addition to *LSU* and *SSU*, several loci such as the internal transcribed spacer (*ITS*), translation elongation factor 1-α (*EF1-α*), RNA polymerase II second largest subunit (*RPB2*), and *β-tubulin* (*TUB*) have been widely used for species identification in *Phoma*-related fungi.

In the present study, the *ITS*, *EF1-α*, and *TUB* sequences of isolate HJ2B3 showed more than 99% similarity to *P*. *radicina* reference sequences available in the NCBI database. Phylogenetic analysis further placed strain HJ2B3 within the *P. radicina* clade, supported by a high bootstrap value (>90%). These findings are consistent with previous studies that identified *P. radicina* using the same molecular markers.

Pathogenicity assays confirmed that *P. radicina* causes root rot in *P. sibiricum*, fulfilling Koch’s postulates. In the inoculated plants, symptoms closely resembling those found in naturally infected specimens were observed. The roots showed clear discoloration, turning dark brown to black, accompanied by tissue necrosis. In addition, the pathogen was successfully re-isolated from the inoculated plants, and its morphological characteristics were identical to those of the original isolate, confirming its pathogenicity.

Our in vitro fungicide sensitivity assay provides baseline data for chemical control of this newly identified pathosystem, based solely on plate assays. The five fungicides tested exhibited distinct inhibitory activities against the pathogen. Difenoconazole, prochloraz, and pyraclostrobin were the most effective, with EC_50_ values of 0.0640, 0.3253, and 0.3602 mg/L, respectively, followed by tetramycin (2.1020 mg/L) and azoxystrobin (5.4280 mg/L). These results were obtained solely from plate assays, representing preliminary laboratory data that require further validation under greenhouse or field conditions.The sensitivity profile observed in this study generally aligns with findings reported for other medicinal plants. The high efficacy of difenoconazole and pyraclostrobin against *P. radicina* in our assay is consistent with their reported strong inhibition of *Fusarium* spp. causing root rot in *Scutellaria baicalensis* and *Bletilla striata* [22,23], while prochloraz exhibited excellent control of *Cylindrocarpon destructans* in ginseng and *Alternaria alternata* in *Saposhnikovia divaricata* [24,25]. In contrast, tetramycin demonstrated only moderate activity against *P. radicina*, despite its reported efficacy against *F. oxysporum*, *F. solani*, and *Sclerotium rolfsii* [20,21], indicating pronounced pathogen-specific variation in sensitivity. Consistent with previous reports on *Polygonatum* species, triazole fungicides, particularly difenoconazole and prochloraz, again demonstrated the highest antifungal efficacy [26,27,38].

*Paraphoma* species have previously been reported to colonize the cysts of *Heterodera glycines* in soybean fields and has been isolated from monocotyledonous hosts across the Iridaceae, Solanaceae, and Rosaceae families [15]. Traditionally *P. radicina* has been regarded as a saprophytic fungus [37]. More recently, it has been identified as a pathogen causing root rot in *Medicago sativa*, resulting in root blackening and necrosis [16]. In the present study, *P. radicina* was consistently isolated from diseased rhizomes of *P. sibiricum* exhibiting root rot symptoms. In vitro sensitivity assays demonstrated that difenoconazole, pyraclostrobin, and prochloraz exhibited significant inhibitory activity against strain HJ2B3.

To date, no reports have documented *P. radicina* as a pathogen of *P. sibiricum*. This study is the first to establish a causal relationship between this fungus and root rot in this host, thereby expanding the known host range of *P. radicina*. Given that *P. sibiricum* is commonly propagated through asexual (clonal) methods, which may increase susceptibility to wound-mediated infection, these findings provide essential baseline data for pathogen identification and inform future disease management strategies for in this important medicinal plant.

## 5. Conclusions

This study identifies *Paraphoma radicina* as the causal agent of root rot in *Polygonatum sibiricum* through morphological and molecular analyses (*ITS*, *EF1-α*, and *TUB*), with pathogenicity confirmed by in vitro inoculation assays, and provides the first baseline data on its in vitro fungicide sensitivity. Among the tested fungicides, difenoconazole and prochloraz exhibited the strongest inhibitory activity, while tetramycin and azoxystrobin showed limited effects. Future work should validate these results under greenhouse and field conditions and explore integrated management strategies for sustainable control of *P. sibiricum* root rot.

## Figures and Tables

**Table 1:** Primers used in this study.

Primer Name	Sequence (5′-3′)	References
ITS1	TCCGTAGGTGAACCTGCGG	White et al., 1990 [30]
ITS4	TCCTCCGCTTATTGATATGC	
TuB2Fd	GTBCACCTYCARACCGGYCARTG	Woudenberg et al., 2009 [31]
TuB4Rd	CCRGAYTGRCCRAARACRAAGTTGTC	
EF1-728F	CATCGAGAAGTTCGAGAAGG	Carbone & Kohn, 1999 [32]
EF2	GGARGTACCAGTSATCATG	O’Donnell et al., 1998 [33]

**Table 2:** Reference isolates and GenBank accession numbers used in this study.

Species	Culture Accession	Location	GenBank Accession Number		
			ITS	TUB	EF1
*Paraphoma radicina*	CBS 102875	Germany	KF251173	KF252668	KF253131
*P. radicina*	CBS 111.79	The Netherlands	KF251172	KT309566	KF253130
*P. radicina*	LYZ 187	China	MH429796	MK404050	MK404051
*P. chlamydocopiosa*	UMPc01	Australia	KU999072	KU999084	KU999080
*P. chlamydocopiosa*	UTAS01	Australia	KX376282	KX376290	KX376298
*P. dioscoreae*	CBS 135100	Republic of Korea	KF251167	KF252662	KF253125
*P. dioscoreae*	CPC 11355	Republic of Korea	KF251168	KF252663	KF253126
*P. dioscoreae*	CPC 11361	Republic of Korea	KF251169	KF252664	KF253127
*P. fimeti*	CBS 170.70	The Netherlands	KF251170	KF252665	KF253128
*P. fimeti*	CBS 164.31	Unknown	KY559063	KY559077	KY559070
*P. fimeti*	CBS 258.68	Germany	KY559064	KY559078	KY559071
*P. pye*	UMPp02	Australia	KU999073	KU999085	KU999081
*P. pye*	UMPp04	Australia	KU999075	KU999087	KU999083
*P. vinacea*	UMPV002	Australia	KU176885	KU176893	KU176897
*P. vinacea*	UMPV003	Australia	KU176886	KU176894	KU176898
*P. vinacea*	UMPV004	Australia	KU176887	KU176895	KU176899
*P. chrysanthemicola*	CBS 172.70	The Netherlands	KF251165	KF252660	KF253123
*P. chrysanthemicola*	CBS 522.66	United Kingdom	KF251166	KF252661	KF253124
*Neosetophoma samarorumc*	CBS 138.96	The Netherlands	KF251160	KF252655	KF253119
*P. radicina*	Not deposited	this study	PX123865	PX128136	PX128137

**Table 3:** Information on the Chemical Agents Tested in the Toxicity Bioassay.

Chemical	Concentration (mg/L)					
	pc1	pc2	pc3	pc4	pc5	pc6
Tetramycin	0	0.60	1.20	1.8	2.1	2.7
Difenoconazole	0	0.04	0.40	4.0	40.0	400.0
Pyraclostrobin	0	0.03	0.30	3.0	30.0	300.0
Prochloraz	0	0.045	0.45	4.5	45.0	450.0
Azoxystrobin	0	0.025	0.25	2.5	25.0	250.0

Note: pc indicates pesticide concentration.

**Table 4:** In Vitro Toxicity of Five Fungicides against *P radicina* Strain HJ2B3 at Different Pesticide Concentrations (pc1–pc6).

Chemicals	Colony Diameter (mm)						Toxicity Regression Equation	R^2^	EC_50_
	pc1	pc2	pc3	pc4	pc5	pc6			
Tetramycin	21.00 ± 1.73 a	16.67 ± 0.58 b	15.67 ± 0.58 bc	14.33 ± 0.58 cd	14.33 ± 0.58 cd	13.00 ± 1.00 d	y = 0.9889x + 4.6808	0.9465	2.10
Difenoconazole	22.67 ± 0.58 a	16.33 ± 1.15 b	11.33 ± 1.15 c	9.33 ± 0.58 d	8.67 ± 1.15 de	7.33 ± 0.58 e	y = 0.5194x + 5.6204	0.9614	0.06
Pyraclostrobin	22.33 ± 0.58 a	17.67 ± 1.15 b	14.67 ± 0.58 c	12.67 ± 1.15 d	9.67 ± 0.58 e	9.33 ± 0.58 e	y = 0.4092x + 5.1819	0.9476	0.36
Prochloraz	24.33 ± 0.58 a	20.33 ± 1.15 b	14.33 ± 0.58 c	11.33 ± 0.58 d	7.33 ± 0.58 e	7.33 ± 0.58 e	y = 0.7488x + 5.3653	0.9418	0.32
Azoxystrobin	23.67 ± 0.58 a	19.33 ± 0.58 b	17.67 ± 0.58 c	15.67 ± 0.58 d	13.67 ± 0.58 e	13.00 ± 1.00 e	y = 0.2615x + 4.8079	0.9788	5.43

Notes: Data under Colony diameter (mm) are expressed as mean ± SD (*n* = 3). Means followed by different letters within a row differ significantly (*p* < 0.05). y: inhibition rate; x: logarithmic value of fungicide concentration; R^2^: correlation coefficient of the regression equation. Different lowercase letters indicate significant differences in colony diameter among concentrations within the same fungicide, determined by post hoc Tukey’s test (*p* < 0.05). (*n* = 3).

## Data Availability

The data presented in this study are available on request from the corresponding authors due to they are part of an ongoing project.

## Supplementary Materials

The following supporting information can be downloaded at: https://www.mdpi.com/article/10.3390/jof11110770/s1, Figure S1: Colony characteristics of HJ2B3 after one week of growth on six different media; Table S1: Types of the test medium and the preparation method. Table S2: Colony growth of strain HJ2B3 on six different culture media.
