# Supplementary material for: Identification, Characterization, and In Vitro Fungicide Sensitivity of Paraphoma radicina, the First Reported Cause of Root Rot in Polygonatum sibiricum
Source: J Fungi (Basel). 2025 Oct 26;11(11):770. doi: 10.3390/jof11110770 (PMC12653157; doi:10.3390/jof11110770)
Supplement: Supplementary file 1 [file jof-11-00770-s001.zip › jof-3894350-supplementary.pdf]

Supplementary: Growth morphology of HJ2B3 on different media

The colonial characteristics of HJ2B3 *Paraphoma radicina* after 7 days of incubation on six different artificial media are as follows:

On PDA, the colony reached a diameter of 25.67 mm. It appeared greyish-white to grey in color, surrounded by a margin of white mycelium. The aerial hyphae grew aggregated towards the center of the colony.On PCA, the colony diameter was 24.67 mm. It exhibited grey aerial hyphae, with a regular and sparse margin. The aerial hyphae had a velvety texture and grew towards the center, forming a raised central area.On PNA, the colony grew to 21 mm in diameter. The colony was white with a regular edge. The white, velvety aerial hyphae were sparse at the margin and grew inward, forming a centra protuberance.On OA, the colony diameter was 25.33 mm. It displayed grey aerial hyphae with a regular and sparse margin, which grew towards the center to form a protuberance. The colony color was light olive green.On MEA, the colony reached a diameter of 24.33 mm. The aerial hyphae were floccose, with a brownish margin. The color transitioned from white at the advancing zone to grey near the center. Brownish-yellow concentric rings were observed on the reverse side.On PCM, the colony diameter was 24.67 mm. The colony was grey in color with a regular edge. The aerial hyphae were sparse overall but denser in the center, with the marginal hyphae appearing greyish-white (Figure S1).

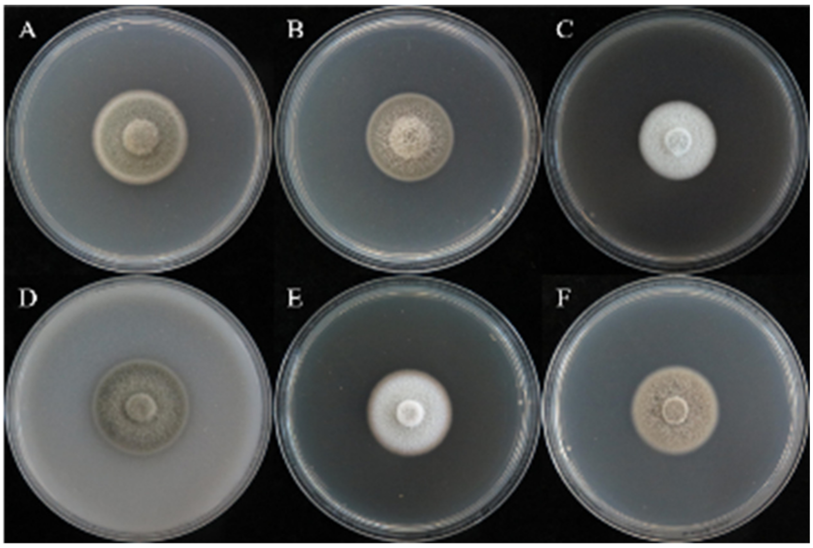

Supplementary Figure S1. Colony characteristics of HJ2B3 after one week of growth on six different media. (A)–(F) correspond to PDA, PCA, PNA, OA, MEA, and PCM, respectively.

Supplementary Table S1. Types of the test medium and the preparation method.

No.	Medium Type	Composition and Preparation Procedure
1	Potato Dextrose Agar (PDA)	200 g potato, 20 g glucose, 17 g agar. Boil peeled, diced potatoes in water for 30 min, filter. Add glucose and agar to filtrate, bring volume to 1 L, and autoclave.
2	Oatmeal Agar (OA)	30 g oatmeal, 15 g agar. Simmer oatmeal in water for 1 hour, filter. Add agar to filtrate, bring volume to 1 L, and autoclave.
3	Pine Needle Agar (PNA)	100 g pine needles, 10 g glucose, 5 g peptone, 1 g K_2HPO_4, 0.5 g $MgSO_4 \cdot 7H_2O$, 18 g agar. Boil pine needles for 1 hour and filter. Add remaining ingredients to filtrate, bring volume to 1 L, and autoclave.
4	Malt Extract Agar (MEA)	30 g malt extract, 3 g peptone, 15 g agar. Dissolve ingredients in distilled water, bring volume to 1 L, and autoclave.
5	Potato Carrot Agar (PCA)	20 g carrot and 20 g potato, 17 g agar. Boil peeled, diced potato and carrot in water for 30 min, filter. Add agar to filtrate, bring volume to 1 L, and autoclave.
6	*Polygonatum sibiricum* Decoction Medium (PCM)	100 g cleaned rhizome of *Polygonatum cyrtonema*, 18 g agar. Cut rhizomes into small pieces, boil for 30 min, and filter. Add agar to filtrate, bring volume to 1 L, and autoclave.

Supplementary Table S2. Colony growth of strain HJ2B3 on six different culture media.

**No.**	**Medium Type**	**Colony Diameter (mm) after 7-day Incubation**
1	PDA	25.67±0.57a
2	PCA	24.67±0.57ab
3	PNA	21.00±0.00c
4	OA	25.33±0.57ab
5	MEA	24.33±0.57b
6	PCM	24.67±0.57ab
